# The Burden of Herpes Zoster on Hospital Admissions: A Retrospective Analysis in the Years of 2015–2021 from the Abruzzo Region, Italy

**DOI:** 10.3390/vaccines12050462

**Published:** 2024-04-26

**Authors:** Piera Scampoli, Giuseppe Di Martino, Fabrizio Cedrone, Camillo Odio, Pamela Di Giovanni, Ferdinando Romano, Tommaso Staniscia

**Affiliations:** 1 Unit of Hygiene, Epidemiology and Public Health, Local Health Authority of Lanciano-Vasto-Chieti, 66100 Chieti, Italy; piera.scampoli@asl2abruzzo.it; 2 Department of Medicine and Ageing Sciences, “G. d’Annunzio” University of Chieti-Pescara, 66100 Chieti, Italy; pamela.digiovanni@unich.it (P.D.G.); tommaso.staniscia@unich.it (T.S.); 3 Unit of Hygiene, Epidemiology and Public Health, Local Health Authority of Pescara, 65100 Pescara, Italy; 4 Hospital Management, Local Health Authority of Pescara, 65100 Pescara, Italy; cedronefab@gmail.com; 5 Digital Health Unit, Department of Health, Abruzzo Region, 65100 Pescara, Italy; camillo.odio@regione.abruzzo.it; 6 Department of Public Health and Infectious Diseases, “La Sapienza” University of Rome, 00100 Rome, Italy; ferdinando.romano@uniroma1.it

**Keywords:** herpes zoster, public health, hospitalizations, epidemiology, vaccines, preventive medicine, Italy

## Abstract

(1) Background: Herpes zoster (HZ) is a disease caused by the reactivation of the Varicella Zoster Virus (VZV). Clinical reactivation, herpes zoster, takes place in 10–20% of subjects who contracted the primary infection, with a higher risk of developing zoster increasing proportionally with age, especially after 50 years of age. HZ is a common clinical problem, particularly among patients aged over 50 years and immunocompromised patients. Immunocompromised patients and adults could present an atypical and more severe course. In addition, they are at greater risk of complications. For this reason, it is important to understand the real burden of the disease and to identify the subjects who are at higher risk of HZ and its complications, also to direct preventive strategies at the right targets. The aim of the present study is to analyze HZ-related hospitalization trends in Abruzzo in the period of 2015–2021. (2) Methods: Data related to hospital admissions were extracted from the hospital discharge records (HDRs) of the whole region, considering all admissions during the years of 2015–2021. The trends in hospital admissions and length of stay were evaluated and analyzed. (3) Results: A total of 768 hospital discharges with a diagnosis of herpes zoster were registered in Abruzzo during the 7-year study period. During the study period, an increasing trend was observed from the year 2015 to the year 2017, ranging from 8.19 cases/100,000 to 11.5 cases/100,000 (APC (Annual percentage change) +20.8%; 95%CI −2.3; 47.6). After the year 2017, a significantly decreasing trend was observed, reaching 5.46 cases/100,000 in the year 2021 (APC −18.4%; 95%CI −31.5; −12.0). Across the entire study period, an average annual percentage change (AAPC) of −7.0% (95%CI −13.0; −1.3) was observed. (4) Conclusions: Despite the trend of a reduction in hospitalizations, this study highlights that HZ continues to have a great impact on public health. So, it is important to update recommendations for the use of the already available HZ vaccine and to implement new strategies to increase awareness of the prevention of the disease.

## 1. Introduction

Herpes zoster (HZ) is a disease caused by the reactivation of the Varicella Zoster Virus (VZV). VZV is an alpha-herpes virus consisting of over 120,000 base pairs of linear double-stranded DNA, and its nucleocapsid is composed of 162 capsomers. The virus is highly cell-associated, and it is only able to infect human cells. During a primary infection, this virus causes a highly contagious disease, Varicella (chickenpox), which mostly affects people during childhood (a higher incidence in the 0–14-year age class), and it is acquired by direct airway contact with respiratory droplets of infected patients or smears from vesicular varicella lesions [1].

VZV resides latently in cells of the dorsal root ganglia, cranial nerve ganglia, various autonomic ganglia in the enteric nervous system, or in astrocytes, and it can be reactivated any time after a primary infection. Herpes zoster, caused by a VZV reactivation, is a vesicular and painful rash, involving one or more adjacent dermatomes. Reactivation develops in 10–20% of patients who contracted the primary chickenpox infection, with a higher risk of developing zoster increasing directly by age, especially after 50 years of age [2]. About 50% of subjects over 80 years report at least one episode of zoster [3]. During a VZV reactivation, VZV virions which have existed in a dormant (latent) state within the ganglia recommence replication and travel to the dermis by anteretrogade axonal transport mechanisms to produce the symptoms of zoster characterized by the appearance of dermatomal skin lesions and vesicles [4]. Lesions are accompanied by localized pain that, in some patients, is so intense and prolonged that it requires the administration of painkiller drugs or local anesthetics. In a number of cases, pain persists for months or years and is characterized by skin hypersensitivity (post-herpetic neuralgia). Special clinical patterns are represented by HZ Ophthalmicus, defined as VZV involvement in the ophthalmic division (V1) of the trigeminal nerve, Ramsay Hunt Syndrome, disseminated HZ, deep HZ, purpuric HZ, and central nervous system HZ [5]. 

In a meta-analysis of the herpes zoster incidence worldwide, a cumulative annual incidence of HZ ranging from 2.9 to 19.5 over 1000 inhabitants and an incidence rate of HZ ranging from 5.23 to 10.9 cases over 1000 person-years were found [6]. The incidence of HZ increased with age and over time [5]. In Italy, 157,000 new cases of HZ are estimated to occur every year. The annual incidence is 6.3/1000 person-years, and 73% of cases affect adult subjects [7]. 

HZ is a common clinical condition, particularly among patients aged over 50 years and immunocompromised patients. Immunocompromised subjects and adults could have atypical and more severe courses and are at a greater risk of complications and recurrence of HZ. HZ and its main complication, post-herpetic neuralgia (PHN), negatively impact a patient’s quality of life [8]. In addition, an HZ infection results in direct and indirect costs and a heavy disease burden, especially in patients of advanced age or with underlying medical conditions [9].

Thus, the epidemiological data, the frequent and serious complications, the impact on the quality of life, and the costs related to the diagnosis and management of HZ patients, including costs related to complications and hospitalizations, are reasons for developing focused preventive measure against this important condition [10]. 

For this reason, it is important to understand the real burden of the disease and to identify the subjects who are at a higher risk of HZ and its complications, also to direct preventive strategies to the right targets.

In Italy, HZ is a disease included in class V in the infectious diseases notification system, and HZ episodes are too often not notified. Thus, the analysis of the hospitalizations is a useful tool for estimating the burden of disease, as performed for several diseases [11]. The HZ-related hospitalizations show the most serious cases, but they reveal a real incidence trend and highlight patients who are at a higher risk of severe disease, towards whom preventive strategies should be addressed as a priority. 

The aim of the present study is to analyze HZ-related hospitalization trends in Abruzzo in the period of 2015–2021 and to identify factors associated with a prolonged length of stay. 

## 2. Materials and Methods

### 2.1. Data Source and Study Design

A retrospective cohort study was performed in Abruzzo, a region in the South of Italy that has about 1.3 million inhabitants. In Abruzzo, healthcare services are divided into four local health authorities (LHAs). Data related to hospitalizations were extracted from the hospital discharge record (HDRs) of the Abruzzo region, considering all hospitalizations registered from the year 2015 to 2021 and also including those registered in other Italian regions of inhabitants from Abruzzo. The HDRs included information on demographic characteristics such as age, gender and residence, a diagnosis-related group (DRG) code used to classify the hospitalization, six possible clinical diagnoses (one principal and up to five secondary conditions), and up to six possible procedures performed during the admission period. Diagnoses and procedures were coded according to the International Classification of Disease, 9th Revision, Clinical Modification (ICD-9-CM).

### 2.2. Inclusion Criteria

In order to find all HZ-related hospitalizations, the following codes were extracted from the HDRs, according to the ICD-9-CM (Table 1).

Thus, all the HDRs with HZ diagnostic codes in primary or in one of the five secondary diagnoses were selected for each year. If a patient had multiple admissions for the same condition, only the first time was included. All following admissions of the same patient presenting HZ codes were excluded. In order to evaluate factors associated with a prolonged length of stay, the comorbidities of each patient were extracted from the HDRs. In particular, all diagnoses included in the Charlson Comorbidity Index (CCI) were considered and extracted according to the algorithm proposed by Quan et al. [12], as reported in Table 2. The Charlson Comorbidity Index was chosen because in the literature, it is identified as a useful tool in identifying the high-risk groups for HZ [13,14].

### 2.3. Statistical Analysis

Continuous variables were reported as mean and standard deviation (SD) or median and interquartile range (IQR) according to their distribution. Categorical variables were reported as frequency and percentage. Comparisons of length of stay among age groups and among types of HZ disease were performed with Kruskall–Wallis rank test. 

A Chi Square test was performed to evaluate the differences between a prolonged length of stay and HZ diagnoses. For each year, annual admission rates were calculated as the ratio between the number of hospitalizations with HZ diagnoses and the Abruzzo resident population per 100,000 inhabitants. Hospitalization rates were standardized for age and gender according to the population of Abruzzo region, registered during the first year of the study (2015). Information about the demographic structure (gender and age) of the Abruzzo population for each year of the study were obtained from the Italian National Institute of Statistics (ISTAT)’s website. The Joinpoint model (Joinpoint version 4.6.0.0, 2018) was performed to estimate time trends of the standardized rates and to calculate the average annual percent change (APC). The APC must be evaluated as a summary measure of the trend over a time interval, which is calculated as a weighted average of the annual percent change. The final model was derived from a linear segment, connected at joint points, which represents the best fit of the observed data. To evaluate factors associated with a prolonged length of stay, the LOS variable was dichotomized, considering a prolonged LOS to be a duration beyond 16 days, corresponding to the upper quartile of the distribution. Factors associated with a prolonged LOS were evaluated with multivariable logistic regression analysis. Results were expressed as odd ratios (ORs) and a relative 95% confidence interval (95%CI). Gender, age, all comorbidities included in the CCI, and HZ types were evaluated for the construction of the regression model. Covariates were assessed using stepwise logistic models before obtaining a final prediction model. The probability for entering the covariates in the model was set at a value of less than or equal to 0.20. A partial likelihood ratio test was performed in order to identify the final prediction model. The goodness of fit of the model was evaluated using the Pearson’s Chi Square estimates.

For all tests, a *p*-value lower than 0.05 was considered significant. The statistical analysis was developed with STATA v18.0 software (StataCorp LLC, College Station, TX, USA).

### 2.4. Ethical Approval

The study was performed according to the data management regulations of the Regional Health Authority of Abruzzo Region and the Italian law on privacy (Art. 20-21 DL 196/2003), published in the Official GAzette, n. 190, on 14 August 2004. The data were encrypted prior to the analysis at the regional statistical office, where a unique identifying code was assigned to each patient. The unique code eliminated the possibility of identifying the patients’ identities. According to Italian law, the use of administrative data does not require any written informed consent from patients. The present study did not directly involve human patients.

## 3. Results

A total of 768 hospital discharges with a primary or secondary diagnosis of herpes zoster were registered in Abruzzo during the 7-year study period. Most of the admissions were ordinary hospitalizations (591, 76.95%), while about a quarter of them were day hospital admissions (177, 23.05%). 

The number of admissions was higher in females (426, 55.47% versus 342, 44.53%), and the mean age of hospitalized patients was 63.22 ± 22.43 years. In total, 286 hospitalizations (37.63% of the total admissions) were recorded for patients who were older than 74 years. During hospitalization, 11 patients died (1.43%), while 602 (78.39%) were discharged directly to their home. The remaining part of patients (155, 20.18%) were transferred to other hospitals or to long-term healthcare facilities. The most represented comorbidities reported in the HDRs were HIV (83, 10.81%), cerebrovascular diseases (74, 9.64%), diabetes (61, 7.94%), and cancer (58, 7.55%), as reported in Table 3.

Among the types of HZ, 36.20% of admissions (n = 278) were HZ without other complications, whereas the remaining 63.8% of the cases were complicated. The most frequent complications were neurological (239 cases, 31.12%), as reported in Table 4. 

The mean annual standardized hospitalization rate for HZ was 8.27 hospital admissions per 100,000 inhabitants. Stratifying by type of admission, although an increase in the number of ordinary admissions occurred in the first three years of the study period, a decrease in the yearly standardized hospitalization rate from 9.00/100,000 in 2017 to 3.90/100,000 at the end of the study period was observed, as reported in Table 5. A decreasing trend was also observed for day hospital admissions: from 2.52 in 2018 to 1.56 in 2021.

**Figure 1:**
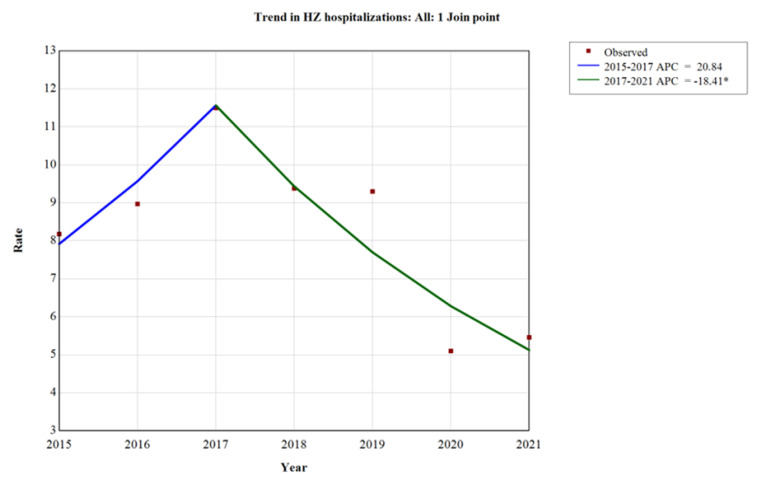
Trend analysis of hospitalizations for HZ, Abruzzo, 2015–2021. * *p* < 0.05.

During the study period, considering all ages, an increasing trend was observed from the year 2015 to the year 2017, ranging from 8.19 cases/100,000 to 11.5 cases/100,000 (APC +20.8%; 95%CI −2.3; 47.6). After the year 2017, a significantly decreasing trend was observed, reaching 5.46 cases/100,000 in the year 2021 (APC −18.4%; 95%CI −31.5; −12.0). Across the entire study period, an average annual percentage change (AAPC) of −7.0% (95%CI −13.0; −1.3) was observed, as shown in Figure 1. 

Stratifying into age groups, the greatest reduction was found among patients who were younger than 30, with an AAPC of −17.2 (95% CI −26.5; −7.6). In particular, an increasing trend was observed between 2015 and 2017, with an APC of 48.4 (95% CI −3.2; 116.5), followed by a decreasing trend between 2017 and 2021, with an APC of −38.1 (95% CI −53.6; −29.2). As reported in Table 6, a reduction in hospitalization rates was highlighted for all age groups, although overall AAPCs were statistically significant only for patients aged between 45 and 59 (AAPC −12.8; 95% CI −19.3; −6.8) and those aged between 60 and 74 (AAPC −7.0; 95% CI −11.0; −2.9). 

**Figure 2:**
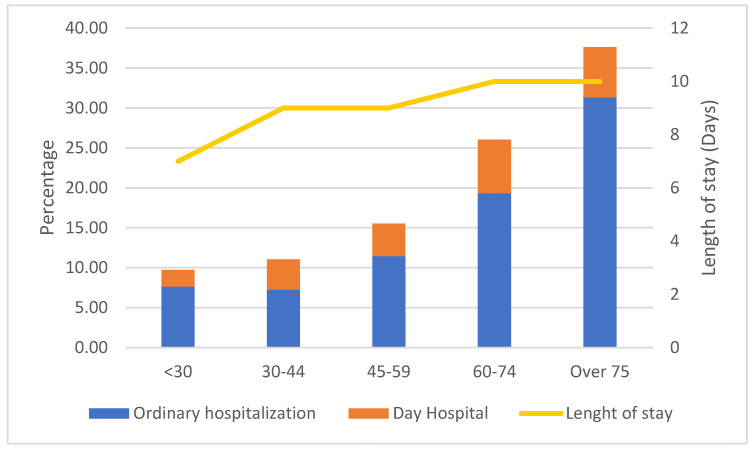
HZ hospitalizations and day hospital admissions stratified by age classes, Abruzzo, 2015–2021.

Hospitalizations for HZ were stratified according to age classes, which revealed an increase with aging, as shown in Figure 2. In the study period, 63.7% of admissions involved patients who were aged more than 60 years (26.1% patients aged 60–74 and 37.6% patients who were older than 74).

Ordinary admissions exhibited a median length of stay of 9 days (IQR 6–16). The length of stay, evaluated only for ordinary hospitalizations, resulted in significantly greater in older patients (*p* = 0.0001), as seen in Figure 2.

Patients with nervous system HZ reported a higher hospital stay (10 days, IQR 6–16), but this was not significantly different in other categories (*p* = 0.139), as shown in Table 7. Nervous system HZ and HZ without other complications were the categories that most frequently led to a prolonged length of stay (*p* = 0.01).

Among the predictors of a prolonged length of stay, an age >74 years (OR 3.09, 95%CI 1.26–7.54), cancer (OR 2.98, 95%CI 1.59–5.60), and HIV (OR 4.59, 95%CI 1.09–19.29) were significant associated factors, as reported in Table 8.

## 4. Discussion

The aim of this study was to describe the impact of HZ in Abruzzo in terms of hospitalizations and to estimate the factors associated with hospitalization and a prolonged length of stay. This study reported an incidence rate of 8.27/100,000, which is similar to other Italian reports (Sicily, 11/100,000) [15] and to other European countries, such as England (8.8/100,000) [16], Denmark (13.1/100,000) [17], and Germany (9.6/100,000) [18]. In the study period, in Abruzzo, a substantial reduction in HZ-related hospitalization rates was observed, confirming a prior national study [15]. This result could be due to a restriction in hospitalization criteria and to a reduction in hospitalizations for all causes in the period of 2020–2021, due to the COVID-19 pandemic, instead of a reduced burden of disease, as reported either for infectious diseases [19] or non-communicable diseases [20]. For these reasons, it is important to conduct further studies to evaluate the hospitalization rates after the pandemic. In fact, a recent study [21] from Spain has highlighted an increase in HZ-related hospitalizations, also likely due to an association between HZ and SARS-CoV-2.

The study confirms that HZ hospitalizations concern elderly individuals, because they report more severe manifestations of the disease due to an age-related decline in cell-mediated immunity to VZV and an increased burden of comorbidities [9,22,23]. In addition, the reduction in hospitalization rates was more marked for younger patients, confirming that older patients remain at a higher risk of HZ-related hospitalization. Also, the length of stay was significantly longer among elderly individuals. Among patients who were aged more than 74 years, the median length of stay was 10 days (95%CI 6–18), whereas it was 7 (95%CI 4–10) among younger patients (under 30).

This point highlights that elderly patients are the most relevant category in terms of the burden of HZ. So, they represent the main target for preventive strategies, such as HZ vaccination. The HZ vaccine was introduced in vaccination schedules of several national immunization plans with the aim of effectively reducing the disease burden. In Italy, in 2014, the ‘Lifetime immunization schedule’ included the recommendation for the use of HZ vaccine in subjects from the age of 50 years and at a higher risk, with comorbidities such as diabetes, cardiovascular disease, chronic obstructive pulmonary disease, or being treated with immunosuppressive agents, and for all persons aged 65 and over [24]. In the first few years, some barriers related to the difficulty of vaccine distribution, the lack of physician recommendations, or the cost of the vaccine hindered the spread of vaccination. The vaccination policies against VZV that have been adopted by many countries are lacking [25] in Italy, and although the HZ vaccination was introduced in the national immunization plan 2017–2019 [26], the coverage objectives are still far from being achieved.

Two types of herpes zoster vaccines are currently available [27]. Vaccines that are now available reduce the incidence of the disease, post-herpetic neuralgia, and the burden of illness [28,29,30,31]. Thus, it is advisable to improve our knowledge of the disease and its complications and also on the usefulness and efficacy of HZ vaccination in order to increase vaccination coverage [32]. Despite these important points of strength, the vaccination campaign probably did not have a strong impact on hospital admission. In Italy, the recombinant vaccine was approved only during the first months of the year 2021. The greater part of admissions were referred to immunocompromised patients that were not able to receive the live-attenuated vaccine. For this reason, the strongest impact on hospital admissions for HZ was due to the COVID-19 pandemic, as was the case for other diseases [16,17].

In addition to aging, factors that are significantly associated with a prolonged length of stay were cancer and HIV, confirming that these comorbidities are triggers for severe episodes of HZ, including disseminated and visceral infections due to impaired cell-mediated immunity [5,33,34]. A prolonged length of stay represents a heavy economic burden for healthcare services, which should be dealt with properly. This point highlights the need to inform healthcare professionals about the opportunity of HZ vaccination in order to directly promote this preventive measure [35].

Definitely, this study strengthens evidence about the great impact of HZ in the elderly and immunocompromised population, identifying it as a primary target for preventive strategies.

### Strengths and Limitations

The main strength of this study was the use of routinely collected data, reporting information on the whole regional population during a seven-year period. In Italy, a specific surveillance system for HZ does not exist. So, the use of HDRs is a useful tool to describe the impact of HZ and its complications, although hospitalizations represent only an aspect of the burden of disease.

The results of this study should be evaluated in light of some limitations. The identification of diagnosis is based on ICD-9-CM codes that do not take into account the severity of the conditions. In addition, the use of administrative data may be limited by the reliability of certain types of information such as drug therapy, clinical information, and laboratory test results. This lack of information may lead to unmeasured confounders hampering the multivariate analysis. Furthermore, an under-reporting of HZ diagnoses in HDRs is possible, leading to an underestimation of the admission rate. In addition, the analysis of risk factors for HZ admission cannot be performed, because HDRs only report data on patients admitted to hospital, and it does not contain information on healthy or non-hospitalized patients.

## 5. Conclusions

Despite the reduction in the hospitalization trend during the years of 2015–2022, this study highlights that HZ continues to have a great impact on public health. The results confirm how advanced age and comorbidities are the risk factors associated with a greater risk of a prolonged length of stay.

Due to population aging, HZ is expected to increase its clinical and economic burden. This is the reason why preventive strategies are important to reduce the incidence and severity of HZ and its complications. Zoster vaccines have been developed and introduced in the Immunization Plan in Italy and in the greater part of European countries. So, it is important to update recommendations for the use of the already available HZ vaccine and to implement new strategies to increase awareness of the prevention of the disease and its complications, both among healthcare professionals and the target population.

## Figures and Tables

**Table 1:** ICD-9-CM codes used to identify HZ-related hospitalizations.

ICD-9-CM Codes	Diagnosis	Diagnosis Group
053.0	HZ with meningitis	HZ with neurological complications
053.1	HZ with other nervous system complications	
053.10	HZ with unspecified nervous system complication	
053.11	Geniculate HZ	
053.12	Post-herpetic trigeminal neuralgia	
053.13	Post-herpetic polyneuropathy	
053.14	HZ myelitis	
053.19	HZ with other nervous system complications	
053.2	HZ with ophthalmic complications	HZ with ophthalmic complications
053.20	HZ dermatitis of eyelid	
053.21	HZ keratoconjunctivitis	
053.22	HZ iridocyclitis	
053.29	HZ with other ophthalmic complications	
053.7	HZ with other specified complications	HZ with other specified complications
053.71	Otitis externa due to HZ	
053.79	HZ with other specified complications	
053.8	HZ with unspecified complications	HZ with other unspecified complications
053.9	HZ without mention of complication	HZ without complications

**Table 2:** Diseases included in Charlson Comorbidity Index and related ICD-9-CM codes.

Diseases	Codes
Cardiovascular diseases	398.91, 402.01, 402.11, 402.91, 404.01, 404.03, 404.11, 404.13, 404.91, 404.93, 425.4–425.9, 428.x
Peripheral vascular diseases	093.0, 437.3, 440.x, 441.x, 443.1–443.9, 47.1, 557.1, 557.9, V43.4
Cerebrovascular disease	362.34, 430.x–438.x
Dementia	290.x, 294.1, 331.2
Chronic pulmonary disease	416.8, 416.9, 490.x–505.x, 506.4, 508.1, 508.8
Rheumatic disease	446.5, 710.0–710.4, 714.0–714.2, 714.8, 725.x
Peptic ulcer disease	531.x–534.x
Diabetes	250.x
Liver diseases	070.22, 070.23, 070.32, 070.33, 070.44, 070.54, 070.6, 070.9, 570.x, 571.x, 573.3, 573.4, 573.8, 573.9, V42.7 456.0–456.2, 572.2–572.8
Hemiplegia or paraplegia	334.1, 342.x, 343.x, 344.0–344.6, 344.9
Kidney disease	403.01, 403.11, 403.91, 404.02, 404.03, 404.12, 404.13, 404.92, 404.93, 582.x, 583.0–583.7, 585.x, 586.x, 588.0, V42.0, V45.1, V56.x
Cancer	140.x–172.x, 174.x–195.8, 200.x–208.x, 238.6 196.x–199.x

**Table 3:** Baseline characteristics of patients admitted for HZ.

	N	%
**Gender**		
Male	342	44.53
Female	426	55.47
**Age**		
<30	74	9.74
30–44	84	11.05
45–59	118	15.53
60–74	198	26.05
>74	286	37.63
**Death**	11	1.43
**Discharged to home**	602	78.39
**Comorbidities**		
HIV	83	10.81
Cerebrovascular diseases	74	9.64
Diabetes	61	7.94
Cancer	58	7.55
COPD	34	4.43
Kidney diseases	33	4.30
Hepatic diseases	31	4.04
Cardiovascular diseases	18	2.34
Rheumatological/Immunomediate diseases	9	1.17
Paraplegia/Hemiplegia	7	0.91

**Table 4:** Types of HZ diagnosis.

	Ordinary Admission n(%)	Day Hospital n(%)	Total n(%)
Nervous System HZ	157(26.57)	82(46.33)	239(31.12)
Ophthalmic HZ	85(14.38)	29(16.38)	114(14.84)
HZ Otitis Externa	11(1.86)	1(0.56)	12(1.56)
HZ with other complications	95(16.07)	30(16.95)	125(16.28)
HZ without other complications	243(41.12)	35(19.77)	278(36.20)

**Table 5:** Number of hospitalizations for HZ and standardized hospitalization rates × 100,000, Abruzzo, 2015–2021.

	Ordinary Admission		Day Hospital		Total	
	n	Standardized Rate (×100,000)	n	Standardized Rate (×100,000)	n	Standardized Rate (×100,000)
**2015**	91	6.83	18	1.35	109	8.19
**2016**	94	6.71	31	2.26	125	8.97
**2017**	120	9.00	34	2.50	154	11.50
**2018**	90	6.86	33	2.52	123	9.38
**2019**	96	7.38	25	1.92	121	9.30
**2020**	50	3.86	16	1.24	66	5.10
**2021**	50	3.90	20	1.56	70	5.46

**Table 6:** Findings of the joint point regression for HZ hospitalization by age group.

Age Groups	Period	APC	95%CI	Overall AAPC	95%CI
**<30**				−17.2 *	−26.5–−7.6
	2015–2017	48.4	3.2–116.5		
	2017–2021	−38.1 *	−53.6–−29.2		
**30–44**				−8.4	−31.3–18.1
	2015–2017	69.2	−26.9–311.3		
	2017–2021	−32.7 *	−71.7–11.6		
**45–59**				−12.8 *	−19.3–−6.8
	2015–2017	7.6	−14.8–35.9		
	2017–2021	−21.5 *	−36.7–−11.9		
**60–74**				−7.0 *	−11.0–−2.9
	2015–2018	13.0 *	2.1–31.5		
	2018–2021	−23.5 *	−34.0–−15.3		
**>74**				−5.9	−21.0–12.2
	2015–2019	2.0	−25.1–38.9		
	2019–2021	−19.9	−69.8–112.8		

* Statistically significant.

**Table 7:** Distribution of lengths of stay by HZ diagnosis.

	Length of Stay Median (IQR)	Prolonged LOS n(%)
Nervous System HZ	10 (6–16)	38(24.20)
Ophthalmic HZ	9 (6–14)	18(21.18)
HZ Otitis Externa	8 (5–11)	1(9.09)
HZ with other complications	7 (5–13)	12(12.63)
HZ without other complications	9 (6–18)	73(30.04)

**Table 8:** Factors associated with prolonged length of stay.

	OR	95%CI	*p*-Value
Gender	1.34	0.89.2.01	0.159
Age			
<30	ref		
30–44	1.35	0.44–4.11	0.590
45–59	2.56	0.97–6.72	0.055
60–74	2.50	0.99–6.32	0.052
>74	3.09	1.26–7.54	0.013
Cancer	2.98	1.59–5.60	0.001
HIV	4.59	1.09–19.29	0.038

## Data Availability

Data are not available due to the restriction policy of the Abruzzo region.

## References

[1] Arvin A.M. (1996). Varicella-Zoster Virus. Clin. Microbiol. Rev..

[2] Freer G., Pistello M. (2018). Varicella-zoster virus infection: Natural history, clinical manifestations, immunity and current and future vaccination strategies. New Microbiol..

[3] Thomas S.L., Hall A.J. (2004). What does epidemiology tell us about risk factors for herpes zoster?. Lancet Infect. Dis..

[4] Zerboni L., Sen N., Oliver S.L., Arvin A.M. (2014). Molecular mechanisms of varicella zoster virus pathogenesis. Nat. Rev. Microbiol..

[5] Patil A., Goldust M., Wollina U. (2022). Herpes zoster: A Review of Clinical Manifestations and Management. Viruses.

[6] van Oorschot D., Vroling H., Bunge E., Diaz-Decaro J., Curran D., Yawn B. (2021). A systematic literature review of herpes zoster incidence worldwide. Hum. Vaccines Immunother..

[7] Gialloreti L.E., Merito M., Pezzotti P., Naldi L., Gatti A., Beillat M., Serradell L., di Marzo R., Volpi A. (2010). Epidemiology and economic burden of herpes zoster and post-herpetic neuralgia in Italy: A retrospective, population-based study. BMC Infect. Dis..

[8] Bader M.S. (2013). Herpes zoster: Diagnostic, therapeutic, and preventive approaches. Postgrad. Med..

[9] Chen P., Chen Z., Xiao Y., Chen X., Li J., Tang Y., Shen M. (2023). Characteristics and economic burden of hospitalized patients with herpes zoster in China, before vaccination. Hum. Vaccines Immunother..

[10] Gabutti G., Franco E., Bonanni P., Conversano M., Ferro A., Lazzari M., Maggi S., Rossi A., Scotti S., Vitale F. (2015). Reducing the burden of Herpes Zoster in Italy. Hum. Vaccines Immunother..

[11] Di Martino G., Di Giovanni P., Cedrone F., D’Addezio M., Meo F., Scampoli P., Romano F., Staniscia T. (2021). The Burden of Diabetes-Related Preventable Hospitalization: 11-Year Trend and Associated Factors in a Region of Southern Italy. Healthcare.

[12] Quan H., Sundararajan V., Halfon P., Fong A., Burnand B., Luthi J.-C., Saunders L.D., Beck C.A., Feasby T.E., Ghali W.A. (2005). Coding Algorithms for Defining Comorbidities in ICD-9-CM and ICD-10 Administrative Data. Med. Care.

[13] D’Hoore W., Bouckaert A., Tilquin C. (1996). Practical considerations on the use of the charlson comorbidity index with administrative data bases. J. Clin. Epidemiol..

[14] Cho S.I., Lee D.H., Park Y.M. (2020). Identification of herpes zoster high-risk group using Charlson comorbidity index: A nationwide retrospective cohort study. J. Dermatol..

[15] Amodio E., Marrella A., Casuccio A., Vitale F. (2022). Decline in hospitalization rates for herpes zoster in Italy (2003–2018): Reduction in the burden of disease or changing of hospitalization criteria?. Aging Clin. Exp. Res..

[16] Hobbelen P.H., Stowe J., Amirthalingam G., Miller L., van Hoek A.J. (2016). The burden of hospitalisation for varicella and herpes zoster in England from 2004 to 2013. J. Infect..

[17] Ultsch B., Siedler A., Rieck T., Reinhold T., Krause G., Wichmann O. (2011). Herpes zoster in Germany: Quantifying the burden of disease. BMC Infect. Dis..

[18] Schmidt S.A., Kahlert J., Vestergaard M., Schønheyder H.C., Sørensen H.T. (2016). Hospital-based herpes zoster diagnoses in Denmark: Rate, patient characteristics, and all-cause mortality. BMC Infect. Dis..

[19] Cedrone F., Montagna V., Del Duca L., Camplone L., Mazzocca R., Carfagnini F., Fortunato V., Di Martino G. (2023). The Burden of Streptococcus pneumoniae-Related Admissions and In-Hospital Mortality: A Retrospective Observational Study between the Years 2015 and 2022 from a Southern Italian Province. Vaccines.

[20] Cedrone F., Di Martino G., Di Giovanni P., Greco E., Trebbi E., Romano F., Staniscia T. (2022). Reduction in Hospital Admissions for Cardiovascular Diseases (CVDs) during the Coronavirus Disease 2019 (COVID-19) Pandemic: A Retrospective Study from a Southern Italian Region in the Year 2020. Healthcare.

[21] Irigoyen-Mansilla V.-M., Gil-Prieto R., Gea-Izquierdo E., Barrio–Fernández J.L., Hernández-Barrera V., Gil-de-Miguel A. (2023). Hospitalization burden related to herpes zoster infection during the COVID-19 pandemic in Spain (2020–2021). Hum. Vaccines Immunother..

[22] Gabutti G., Serenelli C., Cavallaro A., Ragni P. (2009). Herpes zoster associated hospital admissions in Italy: Review of the hospital discharge forms. Int. J. Environ. Res. Public Health.

[23] Levi M., Bellini I., Capecchi L., Pieri L., Bechini A., Boccalini S., Callaioli S., Gasparini R., Panatto D., Tiscione E. (2015). The burden of disease of Herpes Zoster in Tuscany. Hum. Vaccin. Immunother..

[24] Hillebrand K., Bricout H., Schulze-Rath R., Schink T., Garbe E. (2015). Incidence of herpes zoster and its complications in Germany, 2005–2009. J. Infect..

[25] Bonanni P., Azzari C., Castiglia P., Chiamenti G., Conforti G., Conversano M., Corsello G., Ferrera G., Ferro A., Icardi G. (2014). The 2014 lifetime immunization schedule approved by the Italian scientific societies. Italian Society of Hygiene, Preventive Medicine, and Public Health. Italian Society of Pediatrics. Italian Federation of Pediatric Physicians. Italian Federation of General Medical Physicians. Arezzo Service of Legal Medicine. Epidemiol. Prev..

[26] Gabutti G., Valente N., Kuhdari P., Lupi S., Stefanati A. (2016). Prevention of herpes zoster and its complications: From the clinic to the real-life experience with the vaccine. J. Med. Microbiol..

[27] De Waure C., Sisti L.G., Poscia A., Ricciardi W. (2017). The new National Immunization Program 2017–2019 and the Essential Care Levels: What is going to change?. Ig. E Sanita Pubblica.

[28] de Oliveira Gomes J., Gagliardi A.M., Andriolo B.N., Torloni M.R., Andriolo R.B., Puga M.E.D.S., Cruz E.C. (2023). Vaccines for preventing herpes zoster in older adults. Cochrane Database Syst. Rev..

[29] Gabutti G., Bonanni P., Conversano M., Fanelli G., Franco E., Greco D., Icardi G., Lazzari M., Rossi A., Scotti S. (2017). Prevention of Herpes Zoster and its complications: From clinical evidence to real life experience. Hum. Vaccines Immunother..

[30] Izurieta H.S., Wernecke M., Kelman J., Wong S., Forshee R., Pratt D., Lu Y., Sun Q., Jankosky C., Krause P. (2017). Effectiveness and Duration of Protection Provided by the Live-attenuated Herpes Zoster Vaccine in the Medicare Population Ages 65 Years and Older. Clin. Infect. Dis..

[31] Di Giuseppe G., Pelullo C.P., Napoli A., Napolitano F. (2023). Willingness to receive Herpes Zoster vaccination among adults and older people: A cross sectional study in Italy. Vaccine.

[32] Vafai A., Berger M. (2001). Zoster in patients infected with HIV: A review. Am. J. Med. Sci..

[33] Tayyar R., Ho D. (2023). Herpes Simplex Virus and Varicella Zoster Virus Infections in Cancer Patients. Viruses.

[34] Habel L.A., Ray G.T., Silverberg M.J., Horberg M.A., Yawn B.P., Castillo A.L., Quesenberry C.P., Li Y., Sadier P., Tran T.N. (2013). The epidemiology of herpes zoster in patients with newly diagnosed cancer. Cancer Epidemiol. Biomark. Prev..

[35] Curran D., Patterson B.J., Carrico J., Salem A., La E.M., Lorenc S., Hicks K.A., Poston S., Carpenter C.F. (2023). Public health impact of recombinant zoster vaccine for prevention of herpes zoster in US adults immunocompromised due to cancer. Hum. Vaccines Immunother..

